# Safety and preliminary outcomes of short-acting opioid agonist treatment (sOAT) for hospitalized patients with opioid use disorder

**DOI:** 10.1186/s13722-023-00368-z

**Published:** 2023-02-24

**Authors:** Ashish P. Thakrar, Tanya J. Uritsky, Cara Christopher, Anna Winston, Kaitlin Ronning, Anna Lee Sigueza, Anne Caputo, Rachel McFadden, Jennifer M. Olenik, Jeanmarie Perrone, M. Kit Delgado, Margaret Lowenstein, Peggy Compton

**Affiliations:** 1 National Clinician Scholars Program at the Corporal Michael J. Crescenz Veterans Affairs Medical Center, University of Pennsylvania, Philadelphia, USA; 2 Center for Addiction Medicine & Policy, University of Pennsylvania, Philadelphia, USA; 3 Department of Pharmacy, Hospital of the University of Pennsylvania, Philadelphia, USA; 4 Department of Medicine, Perelman School of Medicine, University of Pennsylvania, Philadelphia, USA; 5 School of Nursing, University of Pennsylvania, Philadelphia, USA; 6 Department of Emergency Medicine, Perelman School of Medicine, University of Pennsylvania, Philadelphia, USA; 7 Department of Emergency Medicine, Hospital of the University of Pennsylvania, Philadelphia, USA

**Keywords:** Opioid use disorder, Hospitalized patients, Fentanyl, Opioid agonist treatment, Short-acting opioids, Methadone, Buprenorphine, Opioid withdrawal

## Abstract

**Background:**

Patients with opioid use disorder (OUD) frequently leave the hospital as patient directed discharges (PDDs) because of untreated withdrawal and pain. Short-acting opioids can complement methadone, buprenorphine, and non-opioid adjuvants for withdrawal and pain, however little evidence exists for this approach. We described the safety and preliminary outcomes of short-acting opioid agonist treatment (sOAT) for hospitalized patients with OUD at an academic hospital in Philadelphia, PA.

**Methods:**

From August 2021 to March 2022, a pharmacist guided implementation of a pilot sOAT protocol consisting of escalating doses of oxycodone or oral hydromorphone scheduled every four hours, intravenous hydromorphone as needed, and non-opioid adjuvants for withdrawal and pain. All patients were encouraged to start methadone or buprenorphine treatment for OUD. We abstracted data from the electronic health record into a secure platform. The primary outcome was safety: administration of naloxone, over-sedation, or a fall. Secondary outcomes were PDDs and respective length of stay (LOS), discharges on methadone or buprenorphine, and discharges with naloxone. We compared secondary outcomes to hospitalizations in the 12 months prior to the index hospitalization among the same cohort.

**Results:**

Of the 23 cases, 13 (56.5%) were female, 19 (82.6%) were 40 years or younger, and 22 (95.7%) identified as White. Twenty-one (91.3%) regularly injected opioids and four (17.3%) were enrolled in methadone or buprenorphine prior to hospitalization. sOAT was administered at median doses of 200–320 morphine milligram equivalents per 24-h period. Naloxone administration was documented once in the operating room, over-sedation was documented once after unsanctioned opioid use, and there were no falls. The PDD rate was 44% with median LOS 5 days (compared to PDD rate 69% with median LOS 3 days for prior admissions), 65% of sOAT cases were discharged on buprenorphine or methadone (compared to 33% for prior admissions), and 65% of sOAT cases were discharged with naloxone (compared to 19% for prior admissions).

**Conclusions:**

Pilot implementation of sOAT was safe. Compared to prior admissions in the same cohort, the PDD rate was lower, LOS for PDDs was longer, and more patients were discharged on buprenorphine or methadone and with naloxone, however efficacy for these secondary outcomes remains to be established.

**Supplementary Information:**

The online version contains supplementary material available at 10.1186/s13722-023-00368-z.

## Introduction

Hospitals and emergency departments have become essential sites of care for individuals with opioid use disorder (OUD) [1–4], yet this care is often suboptimal [5]. There are more than 180,000 opioid-related hospitalizations costing more than $2.2 billion in the U.S. every year [6, 7], but less than 15% of patients hospitalized with OUD are discharged on methadone, buprenorphine, or extended-release naltrexone [8, 9], the evidence-based standard of care for OUD in the U.S [10]. More than one in four patients hospitalized for opioid overdose are readmitted within 90 days [11] and 25–30% of patients who inject drugs leave the hospital prematurely as “patient-directed discharges” (PDDs, also known as discharges “against medical advice”) [12]. The consequences of PDDs can be dire: PDDs have been associated with double the odds of 30-day mortality, increased rehospitalizations, and higher care utilization. [13–15]

Patients with OUD report that untreated withdrawal and pain are the two most common reasons for PDDs [16–18]. Patients with OUD also regularly delay acute care because of fear of untreated withdrawal and pain [17, 19]. Guidelines recommend non-opioid adjuvants, methadone, or buprenorphine to treat opioid withdrawal and short-acting opioids for acute pain in patients already treated with methadone or buprenorphine [10, 20]. Each of these approaches can pose challenges. Methadone takes days to weeks of daily dosing to achieve a steady-state therapeutic for withdrawal and cravings [10]; some patients delay or decline buprenorphine due to fear of precipitated withdrawal [21, 22]; and non-opioid adjuvants are rarely effective in isolation [10]. With the rising prevalence of illicit fentanyl, an opioid 25–50 times as potent as heroin [23], and the ensuing elevation in patients’ opioid tolerance [24], some experts have proposed an alternative approach that uses short-acting opioids for the initial treatment of withdrawal and pain while still offering traditional pharmacotherapy for OUD [25–27]. Evidence for the safety and efficacy of this approach, however, is limited.

Two case reports from British Columbia describe the initiation of injectable opioid agonist treatment (iOAT) in the hospital for the purpose of reducing PDDs [28, 29]. In these reports, five patients received intravenous hydromorphone at average doses of 3,000 morphine milligram equivalents (MME) daily. All patients were retained in hospitalized care and successfully transitioned to community-based iOAT with hydromorphone. Currently, iOAT is not authorized in the U.S. although under 21 CFR §1306.07(c), clinicians may legally administer short-acting opioids for intractable pain or to “maintain or detoxify” hospitalized patients as an “incidental adjunct to medical or surgical treatment of conditions other than addiction” [30]. Thus, short-acting opioids in any formulation can be legally administered under these conditions. Although this practice is not new, to our knowledge no prior U.S. studies have described its safety, its effects on the rate of PDDs, or its impact on initiating methadone or buprenorphine.

Here, we report safety and preliminary outcomes from 23 hospitalized patients with OUD who were offered short-acting oral and intravenous opioids titrated to patient-reported relief or tolerability of symptoms, an approach we term “short-acting opioid agonist treatment” (sOAT) for hospitalized patients.

## Methods

### Clinical context

This pilot took place in a quaternary academic medical center in Philadelphia, Pennsylvania that does not have an addiction consult service. Fentanyl was involved in 94% of opioid overdose deaths in Philadelphia in 2020 [31] and, fentanyl or fentanyl analogues were detected in 100% of “heroin” or “dope” samples in the city in the first quarter of 2022. [32]

In summer 2021, the authors and other leaders across the health system developed a quality improvement project to improve care for hospitalized patients with OUD. As part of this project, the authors and health system leaders drafted guidelines for dosing short-acting opioids based on protocols used by local and national peer institutions, existing literature, and consensus from experts in addiction medicine, internal medicine, psychiatry, emergency medicine, pharmacy, pain management, palliative care, and nursing. During guideline development, a pharmacist (TU) with expertise in pain management, palliative care, and substance use disorders piloted implementation by offering interim guidance to clinical teams. The University of Pennsylvania’s Institutional Review Board approved this study as part of the larger quality improvement project. This study was not pre-registered.

### Intervention

The intervention was the implementation of the pilot sOAT protocol in consultation with an expert pain and addiction pharmacist for hospitalized patients with OUD. The protocol consisted of oral oxycodone or hydromorphone scheduled every 4 h (with instructions to hold for sedation) and additional doses of intravenous hydromorphone available as needed. Oral and intravenous doses of short-acting opioids were escalated every 4–12 h, depending on clinical context, for the first 1 to 3 days until patients reported relief or tolerability of pain and withdrawal. Patients who did not respond to this approach were offered patient-controlled analgesia with intravenous hydromorphone. The protocol also included non-opioid withdrawal and pain adjuvants.

Additionally, all patients were encouraged to start methadone or buprenorphine on admission and throughout their hospitalization. Low-dose buprenorphine initiation was available using buccal buprenorphine to aid in transitions from full-agonist opioids [33, 34]. For patients who declined a transition to methadone or buprenorphine, we used shared decision-making to either offer a short-acting opioid taper or abrupt discontinuation prior to discharge. For patients who intend to return to illicit opioid use, an abrupt discontinuation prior to discharge maintains opioid tolerance and might reduce the risk of overdose after discharge [35]. Consistent with 21 CFR §1306.07, no patients were discharged home with short-acting opioids for OUD treatment. As a part of the sOAT protocol, the study pharmacist also reminded clinical teams to discharge all patients with naloxone.

### Cohort selection

During the study period of August 2021 to March 2022, as part of routine clinical care, admitting services asked the study pharmacist for support in managing pain and opioid withdrawal for patients with active OUD. During the study period, the pharmacist and admitting services collaborated to select patients for sOAT based on a global assessment of PDD risk. This risk assessment was based on prior PDDs, the presence of an acute pain indication, or the amount of illicit opioids used (with ≥ 1 “bundle” daily considered high risk). The pharmacist maintained a secure list of all patients offered sOAT per the protocol.

### Outcomes & measures

To describe the intervention, we listed the short-acting opioids used along with total MMEs administered over the first 72 h of the intervention. We also documented use of methadone, buprenorphine, and non-opioid adjuvants.

Our pre-specified primary outcome was “sentinel safety events,” defined as at least one of the following: (1) administration of naloxone; (2) over-sedation, defined as Richmond Agitation Sedation Scale (RASS) [36]  ≤ − 3 or Pasero Opioid-induced Sedation Scale (POSS) [37]  ≥ 3; or (3) a fall. These three indicators were chosen as markers of opioid toxicity based on face-validity and feasibility for this retrospective chart review.

Our pre-specified secondary outcomes were (1) patient-directed discharges (PDDs) and (2) discharges on methadone or buprenorphine maintenance (defined as total daily dose methadone ≥ 40 mg or buprenorphine ≥ 8 mg with a plan to continue after discharge). After preliminary review of the data, we also included a post-hoc analysis of (3) length of stay for all PDDs and (4) patients discharged with naloxone for overdose reversal. We compared secondary outcomes for sOAT cases to outcomes from prior admissions for the same cohort. Specifically, our comparator group consisted of all admissions in the 12 months prior to the index admission for the cohort of sOAT cases. As a sensitivity-analysis, we then limited to only cases with a prior hospitalization in the previous 12 months, comparing their index admission to all 12-month prior admissions.

As exploratory outcomes, we examined maximum and minimum daily pain measured with a 0–10 numerical rating scale and withdrawal measured with the Clinical Opiate Withdrawal Scale (COWS) [38] during the first 3 days of sOAT.

### Data abstraction

Using Microsoft Excel (version 16.64), we created a case review manual (see Additional file 1: Appendix) to systematically collect variables from the electronic medical record (Epic Hyperspace 2017; Epic Systems Corporation, Verona, WI). We defined variables and where to find them for the following categories of interest: patient demographics; past medical, psychiatric, and substance use history; characteristics of the clinical encounter; details of medications administered during hospitalization; and discharge outcomes. This electronic medical record allowed us to view prior and post-discharge hospitalizations for some, but not all, surrounding health care systems.

Four authors (CC, AS, AC, KR) abstracted data from the electronic medical record using REDCap (version 12.5.4), a HIPAA-protected secure platform. Three authors (AW, TU, and APT) verified abstracted data for each case and harmonized conflicting data.

### Data Analysis

We used descriptive statistics to characterize the data. All analyses were conducted using the RStudio (version 1.4.1717) software package.

## Results

We reviewed all 29 cases on the list of patients with OUD offered sOAT that was maintained by the expert pharmacist. Of these, four were never administered short-acting opioids despite recommendations and two were found to have been started on short-acting opioids outside of the sOAT protocol. The final case series cohort comprised the 23 remaining patients.

### Patient & case characteristics

Thirteen (56.5%) patients were female, 19 (82.6%) were younger than 40 years of age, 22 (95.7%) identified as White, none were Hispanic or Latino, 21 (91.3%) were primarily insured by Medicaid, and 14 (60.9%) were documented to have been unstably housed within one year of admission (see Table 1).

**Table 1:** Case characteristics

	Total (%)
	*n* = *23*
Gender (female)	13 (56.5)
Age	
< 30	1 (4.3)
30–39	18 (78.3)
40–49	3 (13.0)
50–59	1 (4.3)
Race	
Black/African-American	1 (4.3)
White	22 (95.7)
Ethnicity	
Not Hispanic or Latino	23 (100.0)
Insurance type	
Medicaid^a^	21 (91.3)
Private	1 (4.3)
Uninsured	1 (4.3)
Unstably housed within the past year	14 (60.9)
Mental health diagnosis	
ADHD	1 (4.3)
Bipolar disorder	4 (17.4)
Depression and anxiety disorders	12 (52.2)
PTSD	3 (13.0)
Other	1 (4.3)
Medical Co-morbidities	
Chronic HCV (untreated)	14 (60.9)
HIV	1 (4.3)
Cirrhosis	1 (4.3)
Chronic kidney disease	1 (4.3)
Substance use disorder diagnosis other than OUD^b^	
Alcohol	5 (21.7)
Benzodiazepines	14 (60.9)
Cocaine	17 (73.9)
Methamphetamine	5 (21.7)
Cannabis	2 (8.7)
Tobacco/Nicotine	18 (78.3)
Daily opioid use	
≤ 14 bags (1 bundle) “dope”	9 (39.1)
15—28 bags (1–2 bundles) “dope”	7 (30.4)
> 28 bags (> 2 bundles) “dope”	7 (30.4)
Intravenous opioid use	21 (91.3)
Xylazine (“tranq”) use	8 (34.8)
Urine drug test results^b^	
Fentanyl	19 (82.6)
Morphine/Codeine	1 (4.3)
Methadone	5 (21.7)
Benzodiazepines	5 (21.7)
Amphetamines	4 (17.4)
Hospital admissions (past 12 mo)	
0	6 (26.1)
1–3	12 (52.2)
≥ 4	5 (21.7)
PDD discharges (past 12 months)	
0	10 (43.5)
1–3	11 (47.8)
≥ 4	2 (8.7)
ED vists without admission (past 12 mo)	
0	11 (47.8)
1–3	10 (43.5)
≥ 4	2 (8.7)
ED visit without admission (past 30 days)	5 (21.7)
Hospital admission (past 30 days)	11 (47.8)
PDD discharge (past 30 days)	9 (39.1)
Acute pain indication (non-exclusive)	
Wound	7 (30.4)
Traumatic injury	1 (4.3)
Skin and soft tissue infection	9 (39.1)
Other	6 (26.1)
None	7 (30.4)
Enrolled in MOUD on admission	
Methadone from OTP	3 (13.0)
Prescribed buprenorphine	1 (4.3)
Not enrolled in MOUD	19 (82.6)
Treated for serious injection-related infection^b^	
Osteomyelitis/discitis	6 (26.1)
Bacteremia/fungemia	5 (21.7)
Endocarditis	4 (17.4)
Septic arthritis	3 (13.0)
Epidural abscess	3 (13.0)
Empyema	1 (4.3)

^a^ Three cases dual-enrolled in medicaid & medicare

^b^ Non-exclusive categories, thus percentages do not add to 100%

Patients had a high rate of charted mental health comorbidities and co-occurring substance use disorders in addition to OUD, including 22 (95.6%) with stimulant use disorder and 14 (60.9%) with benzodiazepine use disorder. Patients used large amounts of opioids daily prior to hospitalization: seven (30.4%) reported using 15–28 bags (1–2 bundles) of heroin or “dope” daily and another seven (30.4%) reported using more than 28 bags (2 bundles) daily. Based on our experience, using at least 1 bundle daily is consistent with very heavy use. Eight patients (34.8%) reported known xylazine use. In Philadelphia during the study period, the primary or secondary opioid in all “heroin” or “dope” samples was fentanyl and xylazine was found in 91% of all illicit opioid samples [32, 39]. On admission, three (13.0%) were enrolled in methadone from an opioid treatment program, one (4.3%) was prescribed buprenorphine, and the remaining 19 (82.6%) were not engaged in pharmacotherapy for OUD.

Patients had high rates of health care utilization in the year prior to the index hospitalization. Twelve (52.2%) had one to three hospitalizations in the past year within local hospitals that used the same electronic medical record system, while five (21.7%) had four or more hospitalizations in the past year. For the index hospitalization, 21 (91.3%) were admitted for a suspected or confirmed infection and 16 (69.6%) had at least one acute pain indication documented on admission.

### Intervention details

Cases received a median of 29 MME (IQR 3–74) over the median 21 h (IQR 12–43) prior to protocol initiation in the form of immediate-release oxycodone, oral hydromorphone, intravenous hydromorphone, intravenous morphine, and/or intravenous fentanyl (see Table 2). Three were continued on methadone, one was continued on buprenorphine, and 10 (43%) were newly administered methadone.

**Table 2:** Intervention details and safety

	Index hospitalization before sOAT^a^	Day 1	Day 2	Day 3
**Short-Acting opioids**	Median (IQR) MME per 24 h	Median (IQR) MME per 24 h	Median (IQR) MME per 24 h	Median (IQR) MME per 24 h
All short-acting opioids	29 (3, 74)	200 (135, 303)	276 (180, 466)	320 (135, 400)
	Cases administered (%)	Cases administered (%)	Cases administered (%)	Cases administered (%)
Oxycodone IR	8 (34.8)	14 (60.9)	14 (60.9)	14 (60.9)
Oxycodone ER	0 (0.0)	2 (8.7)	0 (0)	1 (4.3)
Hydromorphone PO	5 (21.7)	9 (39.1)	8 (34.8)	6 (26.1)
Hydromorphone IV	5 (21.7)	9 (39.1)	6 (26.1)	2 (8.7)
Other short-acting opioids^b^	2 (8.7)	1 (4.3)	1 (4.3)	1 (4.3)
**Medications for OUD**	Cases administered (%)	Cases administered (%)	Cases administered (%)	Cases administered (%)
Buprenorphine	1 (4.3)	1 (4.3)	1 (4.3)	1 (4.3)
Methadone	13 (56.5)	15 (65.2)	15 (65.2)	15 (65.2)
**Non-Opioid adjuvants**	Cases administered (%)	Cases administered (%)	Cases administered (%)	Cases administered (%)
Opioid withdrawal^c^	9 (39.1)	13 (56.5)	14 (60.9)	14 (60.9)
Gabapentinoids	8 (34.8)	13 (56.5)	14 (60.9)	14 (60.9)
Benzodiazepines	7 (30.4)	9 (39.1)	9 (39.1)	8 (34.8)
Barbiturates	1 (4.3)	1 (4.3)	1 (4.3)	1 (4.3)
Non-benzodiazepine anxiolytics^d^	4 (17.4)	4 (17.4)	4 (17.4)	4 (17.4)
Anti-spasmodics	6 (26.1)	13 (56.5)	11 (47.8)	11 (47.8)
NSAIDs or acetaminophen	20 (87.0)	19 (82.6)	19 (82.6)	20 (87.0)
**Safety**	No. (%)	No. (%)	No. (%)	No. (%)
**Naloxone administrations**	0 (0)	1 (4.3)	0 (0)	0 (0)
**Sedation (RASSe ≤ − 3 or POSS‡ ≥ 3)**	0 (0)	0 (0.0)	0 (0.0)	1 (4.3)
**Falls**	0 (0)	0 (0)	0 (0)	0 (0)

^a^ Median (IQR) hours before intervention: 21 (12.0, 43.0)

^b^ Morphine IV, fentanyl IV, remifentanil IV

^c^ Clonidine, ondansetron, or loperamide

^d^ Anti-psychotics or hydralazline

^e^ *RASS* Richmond Agitation Sedation scale, *POSS* Pasero Opioid-induced Sedation Scale, *COWS* Clinical Opiate Withdrawal scale

Over the first 72 h of the sOAT intervention, patients were administered short-acting opioids with a median of 200 MME (IQR 135–303) on Day 1, 276 MME (IQR 180–466) on Day 2, and 320 MME (IQR 135–400) on Day 3. Fourteen cases (60.9%) were administered immediate-release oxycodone, nine (39.1%) received oral hydromorphone, and nine (39.1%) received intravenous hydromorphone. During the first 72 h, no cases received oxycodone concurrently with oral hydromorphone; all nine cases that received intravenous hydromorphone also received either oral oxycodone or oral hydromorphone. During these 3 days, the four cases admitted on methadone or buprenorphine maintenance were maintained on these medications and 12 (52.2%) were newly started on methadone treatment. Median methadone doses for all patients who received methadone increased slightly from 30 mg (IQR 30–45) mg on the first day to 40 mg (IQR 30–50) on the third day of the intervention.

Seventeen (73.9%) patients were tapered off short-acting opioids prior to discharge while six (26.1%) were abruptly discontinued on discharge. Among patients tapered off short-acting opioids, 14 (60.9%) were transitioned to methadone maintenance and two (8.7%) started low-dose buprenorphine during the short-acting opioid taper. One (4.3%) patient was tapered off short-acting opioids and declined transition to medications for OUD after developing withdrawal during a buprenorphine low-dose initiation. Among the six patients who had short-acting opioids abruptly discontinued at discharge, all left as PDDs, five (21.7%) were offered but declined medications for OUD, and one (4.3%) was in the process of initiating methadone treatment.

Non-opioid analgesic adjuvants, gabapentinoinds, and opioid withdrawal adjuvants such as clonidine, ondansetron, and loperamide were administered regularly. Benzodiazepines and antispasmodics were also administered to more than one-third of patients concurrently with short-acting opioids to manage opioid or xylazine withdrawal symptoms and anxiety [40].

### Safety

One patient was documented to have received naloxone during the intervention period (see Table 2). This was noted in the medication administration record while the patient was in the operating room undergoing chest tube placement. Clinical notes do not mention naloxone administration, opioid overdose, or over-sedation at that time, so it is unclear whether naloxone was actually administered or just removed from the automated medication dispensing system.

One event of over-sedation was documented with a POSS of 3 and RASS -1. Scheduled hydromorphone was subsequently held. Per clinical notes, twelve hours later a used syringe was found in the patient’s bed and the patient acknowledged unsanctioned opioid use.

No falls occurred for any patient during the intervention.

### Secondary outcomes

Sixteen of the 23 case patients had been hospitalized within the 12 months prior to the sOAT index admission. These 16 patients had 42 total admissions, none of which included sOAT. We descriptively compared secondary outcomes between all 23 sOAT index admissions and the comparator group of 42 prior admissions in the same cohort.

Ten (43.5%) of the 23 sOAT index admissions ended with a PDD compared to 29 (69.0%) of the 42 prior admissions without sOAT (see Table 3). Among the sOAT index admissions, length of stay for PDDs was 5.0 days (IQR 2.5–8.3) compared to 3.0 days (IQR 1.3–4.2) for PDDs during prior admissions.

**Table 3:** Secondary outcomes

	Prior admissions among the 16 cases with an prior admission in the past 12 months (%)	Index admissions among the 16 cases with an prior admission in the past 12 months (%)	Index admissions among the entire case series cohort (%)
	*n* = *42 admissions*	*n* = *16 admissions*	*n* = *23 admissions*
Patient-directed discharge (PDD)	29 (69.0)	7 (43.8)	10 (43.5)
Length of stay for PDDs	3.0 days [IQR 1.3–4.2]	5.0 days [IQR 3.0–12.5]	5.0 days [IQR 2.5–8.3]
Discharged on medications for OUD	14 (33.3)	11 (68.8)	15 (65.2)
Discharged with naloxone	8 (19.0)	11 (68.8)	15 (65.2)

On index admissions with sOAT, 15 patients (65.2%) were discharged on medications for OUD compared to 14 patients (33.3%) on prior admissions without sOAT. Of the 15 index cases discharged on medications for OUD, 10 (43.4%) were newly started on methadone maintenance, one (4.3%) was newly started on buprenorphine maintenance, three (13.0%) were continued on methadone, and one (4.3%) was continued on buprenorphine from before hospitalization. The single patient newly started on buprenorphine was initiated using low-dose initiation after the first 3 days of sOAT, and thus is not represented in Table 2. Three patients administered methadone during the first 72 h of sOAT were discharged without methadone maintenance; two declined methadone maintenance and one left as a PDD before methadone reached a therapeutic dose. Among the 23 index cases, 15 (65.2%) were discharged with naloxone for overdose reversal compared to 8 (19.0%) during prior admissions without sOAT.

Secondary outcomes were similar when we compared all 42 prior admissions to just the 16 index cases with prior admissions as a sensitivity analysis (see Table 3).

Patients reported high levels of pain throughout the 3 days of sOAT, with median maximum numerical rating scale of 8 throughout the first 3 days of the intervention. Median minimum pain scores rose slightly from 6 (IQR 3–8) on Day 1 to 7 (IQR 4–8) on Day 2, then fell to 5 (3–7) on Day 3; all three of these minimum pain scores were lower than scores reported prior to sOAT initiation (median 7, IQR 1–8). Median maximum and minimum COWS scores fell over the 3 days from a maximum of 8 (IQR 4–10) and minimum of 3 (IQR 1–5) on Day 1, to a maximum of 4 (IQR 2–6) and a minimum of 2 (IQR 1–2) on Day 3.

## Discussion

In this case series of 23 hospitalized patients with OUD, short-acting opioids administered at median daily doses of 200–320 MME (equivalent to 20–35 mg of immediate-release oxycodone every four hours) appeared to be safe. Naloxone administration was documented once in the operating room, over-sedation occurred once after unsanctioned opioid use, and no falls were noted. For our secondary outcomes, the PDD rate was 44% with median LOS 5 days (compared to a PDD rate 69% with median LOS 3 days for prior admissions without sOAT over the 12 months prior), 65% of patients were discharged on buprenorphine or methadone (compared to 33% in prior admissions), and 65% were discharged with naloxone (compared to 19% in prior admissions).

The goal of this study was to describe the safety of this implementation of sOAT for hospitalized patients with OUD. Although it was beyond the scope of this study to collect safety data for the comparator group of 42 prior hospitalizations, safety outcomes from index sOAT admissions did not show definitive evidence of iatrogenic overdose. In this pilot, dosing was guided by a clinical pharmacist with extensive training in and experience with addiction medicine, pain management and palliative care; in other settings, addiction consult services guide dosing for similar approaches [28, 29]. Many hospitals lack this expertise and do not fund clinical services dedicated to patients with substance use disorders [2]. Recent clinical guidelines for managing infective endocarditis from the American Heart Association place the onus of locating these addiction services on health care systems, arguing that failing to provide patients with evidence-based care could place hospitals in violation of the Americans with Disabilities Act [41]. Hospitals must invest in the integration of addiction treatment services to ensure the complex medical needs of patients with SUD are fully and capably met.

Our secondary outcomes, intended to be hypothesis-generating, were encouraging. The PDD rate with sOAT was lower than the PDD rates for hospitalizations without sOAT over the 12 months prior, although PDDs still occurred in 10 cases. This suggests a confluence of factors. First, it may be a sign of underdosing. Our short-acting opioid doses were approximately one-tenth those used in the two case reports of a similar approach from British Columbia, although comparisons are imperfect due to differences in the illicit drug supply and our concomitant use of methadone or buprenorphine [28, 29]. In our cases, pain and opioid withdrawal scores were lower on the third day of sOAT but remained non-zero. We did not dose short-acting opioids to a goal COWS or pain score because, in our experience, these scores often did not match patients’ subjective reports symptom tolerability. Second, polysubstance use—whether known, intentional, or neither—was common. Xylazine, benzodiazepine, or stimulant withdrawal may have contributed to PDDs. Third, reasons for leaving the hospital are not limited to pain and withdrawal. Other studies have shown that patients with substance use disorders also leave early due to stigma, discrimination, and frustration with hospital restrictions, none of which were directly addressed with sOAT [16]. Last, the PDD rate also reflects the high baseline risk of PDDs in the patients selected for this pilot. In this context, it is encouraging that median length of stay for PDDs was longer than during prior hospitalizations.

Fifteen patients were discharged on methadone maintenance and two were discharged on buprenorphine. This includes ten new initiations of methadone and one new initiation of buprenorphine. At this point, the only evidence available to guide initiation of methadone for hospitalized patients are case reports; all other evidence is derived from outpatient settings among individuals who were using opioids other than fentanyl. Low-dose initiation of buprenorphine is currently only supported by case series and uncontrolled, retrospective cohort studies. Future research will need to explore the optimal way to transition hospitalized patients from short-acting opioids to these maintenance medications for OUD.

This study has limitations. First, this was a retrospective case series at a single hospital without a control group, thus safety outcomes may not generalize to other settings and our secondary outcomes should be considered hypothesis-generating. Second, our sample of patients lacked racial and ethnic diversity, with 95.7% of patients self-identified as White and all identified as non-Hispanic/Latino. Future work will need to ensure that sOAT for hospitalized patients is evaluated in diverse cohorts and that implementation does not perpetuate or exacerbate existing racial disparities in acute pain management [42]. Third, our review of prior hospitalizations only captured the subset local hospital systems that used the same electronic medical record as our health system. Fourth, this approach relied on scheduled medications every four hours in addition to as-needed adjuvants, which might be challenging for nurses to administer in certain contexts. Last, this case series did not capture the patient experience of the intervention. Future work should build on this study prospectively with a diverse cohort, and supplement quantitative outcomes with qualitative evidence from patients and clinicians.

## Conclusion

In this case series of 23 high-risk patients with OUD, sOAT for hospitalized patients, an approach to treating withdrawal and pain with oral and intravenous formulations of short-acting opioids, was found to be safe. Secondary outcomes were also encouraging and deserve further investigation: the rate of patient-directed discharges (PDDs) was lower than the rate from prior hospitalizations though remained high, and two-thirds of cases were discharged on methadone and buprenorphine maintenance with naloxone. Efficacy with respect to these outcomes as well as the impact of sOAT on pain and withdrawal remain to be established. As fentanyl becomes further entrenched in the supply of illicit opioids across North America, larger, prospective, controlled studies will be needed to evaluate sOAT and its implementation across a broad range of hospitals.

## Supplementary Information

**Additional file 1**. Case Review Manual.

## Data Availability

Data abstraction form included as an Additional file 1: Appendix.

## Abbreviations

OUD: Opioid use disorder
sOAT: Short-acting opioid agonist treatment
PDD: Patient-directed discharge
MME: Morphine milligram equivalents
IQR: Interquartile range
COWS: Clinical Opiate Withdrawal Scale
RASS: Richmond Agitation Sedation Scale
POSS: Pasero Opioid-induced Sedation Scale
