# Supplementary material for: Safety and preliminary outcomes of short-acting opioid agonist treatment (sOAT) for hospitalized patients with opioid use disorder
Source: Addict Sci Clin Pract. 2023 Feb 24;18:13. doi: 10.1186/s13722-023-00368-z (PMC9951406; doi:10.1186/s13722-023-00368-z)
Supplement: Supplementary file 1 — Additional file 1. Case Review Manual. [file 13722_2023_368_MOESM1_ESM.docx]

**Short-Acting Opioids Case Series – Chart Review Manual**

*Last revised – May 26, 2022*

***Patient Demographics***

Variable: MRN

Location in chart: Left-hand column

If data missing: skip

_____________________________________________________________________________

Variable: DOB

Location in chart: Left-hand column

If data missing: skip

_____________________________________________________________________________

Variable: Age on date of presentation

Location in chart: Left-hand column

Procedure: Calculate age if patient has had a birthday between admission & day of chart review

If data missing: skip

____________________________________________________________________________

Variable: Gender

Location in chart: Left-hand column

If data missing: skip

____________________________________________________________________________

Variable: Race

Location in chart: Left-hand column, hover over “legal name”

If data missing: skip

_____________________________________________________________________________

Variable: Ethnicity

Location in chart: Left-hand column, hover over “legal name”

If data missing: skip

_____________________________________________________________________________

Variable: Preferred language

Location in chart: Left-hand column, hover over “legal name”

If data missing: skip

_____________________________________________________________________________

Variable: Houseless at time of presentation?

Location in the chart: Search “Homeless”. Mark as yes if homelessness documented within past year.

If data missing: skip

_____________________________________________________________________________

Variable: Insurance

Location in chart: Left-hand column, “primary coverage”

If data missing: skip

_____________________________________________________________________________

***Medical & Psychiatric History***

Variable: Documented Medical Co-Morbidities

Location in chart: Epic search

Procedure:

- Search for the following terms: “HIV,” “hepatitis C”, “HCV”, “CKD”, “ESRD”, “cirrhosis”
- Mark yes if any of these are documented over the past 5 years.

_____________________________________________________________________________

Variable: Documented Psychiatric Co-Morbidities

Location in chart: Epic search

Procedure:

- Search for the following terms: “depression”, “anxiety”, “panic attack”, “OCD”, “PTSD”, “bipolar”, “schizo”, “ADHD”
- Mark yes if any are documented over the past 5 years

_____________________________________________________________________________

Variable: Other SUD Diagnoses

Location in chart: Epic search

Procedure:

- Search for the following terms: “Alcohol use disorder”, “alcohol dependence,” “alcohol withdrawal,” “benzodiazepine use disorder,” “benzodiazepine dependence,” “benzodiazepine withdrawal,” “cocaine use disorder,” “cocaine dependence,” “cocaine withdrawal,” “methamphetamine use disorder,” “methamphetamine dependence,” “methamphetamine withdrawal,” “cannabis use disorder,” “cannabis dependence,” “cannabis withdrawal,” and “smoker”
- **Please confirm that the presence of one of these is actually documented (sometimes clinicians write “no alcohol withdrawal”, and this would not count of course)**.
- Mark yes if any are documented over the past five years.

_____________________________________________________________________________

Variable: # of prior hospital admissions in past 12 months

Location: Chart Review > Encounters > check-off “Admissions”

Procedure:

- Count hospitalizations for 12mo prior to index hospitalization

_____________________________________________________________________________

Variable: Prior hospitalization: a) Within 30d of admission

Location: Chart Review > Encounters > check-off “Admissions”

Procedure:

- Calculate whether admission occurred within 30 days of presentation for index hospitalization

_____________________________________________________________________________

Variable: Prior hospitalization: b) LOS

Location: Chart Review > Encounters > click on specific Admission

Procedure:

- 1. Look at discharge summary (should pop up in a window when you click on the admission)
- 2. Find Length of Stay (or LOS).

_____________________________________________________________________________

Variable: Prior hospitalization: c) Patient-Directed Discharge

Location: Chart Review > Encounters > click on specific Admission

Procedure:

- 1. Look at discharge summary (should pop up in a window when you click on the admission)
- 2. Search the discharge summary for “AMA” or “Against Medical”
- 3. If patient left AMA, read note to see if reason is listed

_____________________________________________________________________________

Variable: Prior hospitalization: d) Discharged on MOUD

Location: Chart Review > Encounters > click on specific Admission

Procedure:

- 1. Look at discharge summary (should pop up in a window when you click on the admission)
- 2. Search the discharge summary for “buprenorphine”, “methadone”, and “naltrexone”
- 3. Document if patient was discharged on buprenorphine or methadone maintenance with plan to continue after discharge

***Characteristics of Clinical Encounter***

***** Location for all of these will be Chart Review > Encounters tab > right-click on the index admission and select “Edit Encounter” *****

_____________________________________________________________________________

Variable: Opioid(s) used and amount used daily

Location: H&P note

_____________________________________________________________________________

Variable: Injecting drugs

Location: H&P note. Might be listed as “IV” use

_____________________________________________________________________________

Variable: Xylazine (“tranq”) use

Location: 1. H&P note, 2. Search for “xylazine” or “tranq” and mark if use was noted elsewhereduring the index admission

_____________________________________________________________________________

Variable: Results of urine drug testing

Location: 1. H&P note, 2. Search “urine tox” for results from index admission

_____________________________________________________________________________

Variable: Engaged in MOUD prior to admission

Location: H&P note. If not clear, you can try searching the note for “buprenorphine,” “methadone,” or “naltrexone”

_____________________________________________________________________________

Variable: Admission diagnosis

Location: H&P note. ***

_____________________________________________________________________________

Variable: Admitting team

Location: H&P note.

_____________________________________________________________________________

Variable: Acute pain & reason for acute pain

Location: H&P note.

***Intervention Days 0, 1, 2, and 3***

***** Location for all of these will be Chart Review > Encounters tab > right-click on the index admission and select “Edit Encounter” *****

_____________________________________________________________________________

Variable: Day 0 definition

Location: Notes > search “Tanya Uritsky”

Procedure:

1. Look for Tanya’s note and recommendations.
2. Go to the “Benzo/Opioid” tab and find when these recommendations were implemented.
3. Calculate hours between ED presentation and implementation of Tanya’s recommendations

_____________________________________________________________________________

Variable: Use of short-acting opioids, total mg administered, MMEs, MOUD, non-opioid adjuvants, naloxone

Location: Benzo/Opioid tab

_________________________________________________________________________

Variable: Sedation, pain, withdrawal scores

Location: Benzo/Opioid tab

_________________________________________________________________________

Variable: Treatment of alcohol or benzo withdrawal

Location: Benzo/Opioid tab

Procedure:

1. Look for CIWA-Ar measurements (at least 3)
2. Look for administration of benzodiazepines

***Intervention Day 3***

_________________________________________________________________________

Variable: Was a fall documented in the chart during Days 0-3?

Location: Search

Procedure: 1. Search for “fall”, document only if fall happened (nurses documenting “fall risk assessment completed” don’t count)

_____________________________________________________________________________

Variable: Self-administered street drugs

Location: Search

Procedure:

1. Search for “used drugs”
2. Search for “self-administered”
3. Document only if note indicates confidence about drug use.

***Hospital Outcomes***

***** Location for all of these will be Chart Review > Encounters tab > right-click on the index admission and select “Edit Encounter” *****

_________________________________________________________________________

Variable: Teams consulted

Location: Notes > Consults

_________________________________________________________________________

Variable: Serious injection-related infections treated during hospitalization

Location: Discharge summary

Procedure:

1. Read DC summary or search the DC summary for these infections

_________________________________________________________________________

Variable: Discharged on medications for opioid use disorder (methadone, buprenorphine, or XR-naltrexone)

Location: Discharge summary

Procedure:

1. Read DC summary and assess whether these medications were continued. Methadone often will not appear in the discharge meds, but the DC summary should list where/if patient will continue methadone the day following discharge. Buprenorphine will usually be prescribed on discharge with a “bridge” script. Please text/email me (Ashish) if you are not sure and I can help assess!

_________________________________________________________________________

Variable: Discharged with naloxone

Location: Discharge summary

Procedure:

1. Read DC summary and assess. It will usually be in the discharge medications, but sometimes it might be handed to patient and just documented in the text of the DC summary. **Note that buprenorphine-naloxone is a buprenorphine prescription co-formulated with naloxone and does not count as a naloxone prescription**.

_________________________________________________________________________

Variable: Discharge disposition

Location: Discharge summary

_________________________________________________________________________

Variable: Patient-directed discharge

Location: Discharge summary

Procedure:

1. Search the discharge summary for “AMA” or “Against Medical”
2. If patient left AMA, read the DC Summary to see if the reason is listed

_________________________________________________________________________

Variable: Length of stay

Location: Discharge summary

***Post-Discharge Outcomes (up to 30 days post-discharge)***

_________________________________________________________________________

Variable: Number of ED presentations up to 7 days after discharge

Location: Encounters tab

_________________________________________________________________________

Variable: Number of hospital admissions up to 7 days after discharge

Location: Encounters tab

_________________________________________________________________________

Variable: Number of ED presentations up to 30 days after discharge

Location: Encounters tab

_________________________________________________________________________

Variable: Number of hospital admissions up to 30 days after discharge

Location: Encounters tab

_________________________________________________________________________

Variable: Deceased at time of chart review

Location: Left-hand column (it’s obvious)
